# The PPAR**α** Agonist Fenofibrate Reduces Prepulse Inhibition Disruption in a Neurodevelopmental Model of Schizophrenia

**DOI:** 10.1155/2012/839853

**Published:** 2012-05-15

**Authors:** Benjamin Rolland, Kevin Marche, Olivier Cottencin, Régis Bordet

**Affiliations:** ^1^Département de Pharmacologie Médicale, EA 1046, Université Lille Nord de France, 1 place de Verdun, 59000 Lille, France; ^2^Service de Psychiatrie et Addictologie, CHU Lille, 59037 Lille, France; ^3^LNFP, EA4559, Université Lille Nord de France, 59000 Lille, France

## Abstract

Oxidative stress has been implicated in neurodevelopmental theories of schizophrenia. Antioxidant Peroxysome Proliferator-Activated Receptors *α* (PPAR*α*) agonist fenofibrate has neuroprotective properties and could reverse early preclinical infringements that could trigger the illness. We have evaluated the neuroprotective interest of fenofibrate in a neurodevelopmental rat model of schizophrenia. The oxidative lesion induced by Kainic Acid (KA) injection at postnatal day (PND) 7 has previously been reported to disrupt Prepulse Inhibition (PPI) at PND56 but not at PND35. In 4 groups of 15 male rats each, KN (KA-PND7 + normal postweaning food), KF (KA-PND7 + fenofibrate 0.2% food), ON (saline-PND7 + normal food), and OF (saline + fenofibrate food), PPI was recorded at PND35 and PND56. Three levels of prepulse were used: 73 dB, 76 dB, and 82 dB for a pulse at 120 dB. Four PPI scores were analyzed: PPI73, PPI76, PPI82, and mean PPI (PPIm). Two-way ANOVAs were used to evaluate the effects of both factors (KA + fenofibrate), and, in case of significant results, intergroup Student's *t*-tests were performed. We notably found a significant difference (*P* < 0.05) in PPIm between groups KN and KF at PND56, which supposes that fenofibrate could be worthy of interest for early neuroprotection in schizophrenia.

## 1. Introduction

Schizophrenia is a chronic and severe mental illness that affects around 1% of the population and is characterized by delusions, hallucinations, and thought disorder [1]. Two different types of pathophysiological processes have been suggested to underlie schizophrenia: neurodegeneration and neurodevelopmental disruption [2]. Neurodegenerative models suppose that lesional mechanisms and neuronal death are continuous, which could underlie some progressive deficits observed during the course of the illness [3]. Neurodevelopmental models suppose that some early neuronal infringements could disrupt the normal course of the cerebral development, leading to prodromal abnormalities and finally much later to the occurrence of the first clinical symptoms, which will sign the onset of the illness [4].

The concept of neuroprotection applied to schizophrenia could be considered differently in regard to these two models. In a neurodegenerative perspective, neuroprotective therapeutics should be quickly applied after the onset of the illness in order to limit the extension of lesions and consequently the aggravation of symptoms and deficits [5]. On the other hand, neuroprotective strategies could be also envisaged during or between the initial infringement and the occurrence of symptoms, which could limit the long-term symptom burden or even prevent the outcome of the illness.

Oxidative stress has been suggested to be a possible mechanism that could be involved in both neurodevelopmental and neurodegenerative hypotheses of schizophrenia [6, 7]. In rodent, perinatal oxidative stress injuries trigger delayed-onset cognitive dysfunctions, similar to those found in patients [6, 8]. More precisely, lesions made at postnatal day 7 (PND7) induce disruptions in the neurodevelopment of hippocampus that are responsible for later dysfunctions in a specific cognitive parameter called prepulse inhibition (PPI) [9]. PPI is the attenuation of the startle reflex when the startling stimulus is shortly preceded by a weaker, nonstartling sensory stimulus (prepulse) [10]. Neonatal oxidative lesions induce reduction of PPI scores that occur only after puberty [8, 9], like observed in patients with schizophrenia [11]. The intraperitoneal injection of pro-oxidative drug kainic acid (KA, 1.5 mg/kg) at PND7 reduces PPI at PND56 (postpubertal age) but not at PND35 (prepubertal age) [12].

As previously mentioned, the perspective of developing disease-modifying therapeutics that could be delivered to patients at the very onset of schizophrenia, or even during phases of neurodevelopmental injuries, is becoming one of the major topics of current and future research of the field [5]. Treatments that reverse oxidative stress could improve the symptomatic and functional outcome of patients and reverse the natural course of the illness [6]. N-Acetyl-Cysteine, a glutathione-peroxidase precursor that has antioxidant properties, has been tested in both preclinical and clinical studies and has shown promising results in both humans and animals, in restoring several types of cognitive alterations [13, 14].

Peroxysome Proliferator-Activated Receptors *α* (PPAR*α*) could be other interesting targets for reducing oxidative stress in schizophrenia. PPAR*α* are nuclear receptors whose activation regulates the gene expression of major cell metabolism pathways, including energy combustion, hepatic steatosis, lipoprotein synthesis, and inflammation [15]. PPAR*α* agonist fenofibrate reduces oxidative stress processes in both rodents and humans [16, 17]. Fenofibrate has shown neuroprotective action in animal models of stroke and Huntington's disease [18, 19]. Moreover, fenofibrate can reverse the cognitive dysfunctions that are neurodevelopmentally induced by ethanol in fetal rat [20].

In the present study, we have tested the neuroprotective effects of fenofibrate on the pre-cited model, based on KA-induced delayed alterations of PPI in rat [12].

## 2. Materials and Methods

### 2.1. Animals

60 male rat pups were obtained from 18 time-mated Wistar females (Janvier, Le Genest Saint Isle, France). Females were housed individually in standard maternity cages with continuous access to drinking fluid and food. The colony room had a 12 h light/12 h dark cycle with lights on at 6 AM. The day of birth of the pups was designated PND 0. On PND 3, the animals were sexed to keep only 4 males per litter (or least if not possible), in order not to mix pups of the same litter in the same group (see what follows). All experiments were performed in accordance with the current French and European Union legislation on animal experimentation.

### 2.2. Kainic Acid Injection

On PND 7, rat pups were removed from the litter, weighed, and individually placed in small glass boxes for intraperitoneal injection. KA (1.5 mg/kg, Sigma-Aldrich) or saline was injected with a 30-gauge needle (10 mL/kg). 2 animals from each litter received KA and 2 others received saline. There were 6 deaths out of 66 rats, and all pups that died had received KA. These deaths were the consequence of the seizures, which is a usual effect induced by KA [12]. Pups that survived the injection were earmarked according to treatment condition and were returned to their mother. The litters were then left undisturbed weaning on PND 25. In the end were kept 30 male rats injected with KA, and 30 male rats injected with saline.

### 2.3. Fenofibrate Administration and Groups

On PND 25, rat pups were separated from their mother and housed in cages of three to five animals. Half of the animals were fed with a diet containing 0.2% fenofibrate (UAR, Villemoisson-sur-Orge, France). The other half of animals were fed with the same diet but without fenofibrate. Care was taken to ensure that animals of each litter were separated among the four following groups:

- Group KN: KA at PND7 and normal food after weaning on PND25,
- Group KF: KA at PND7 and fenofibrate 0.2% after PND25,
- Group ON: saline injection at PND7 and normal food after PND 25,
- Group OF: saline injection at PND7 and fenofibrate 0.2% after PND25.

The fenofibrate dose was chosen according to our previous experimental findings in brain disorder models [18, 20]. All rats were regularly handled from PND25.

### 2.4. Prepulse Inhibition

On PND 35 and 56, rats were taken individually to the PPI apparatus (*LE 118-8 Startle and Fear Interface, Panlab, Barcelona, Spain)*. A sound-attenuated startle chamber contained a clear Plexiglas cylinder resting on a piezoelectric transducer that detected the vibrations caused by the animals' movements. A computer was used to control the timing and presentation of acoustic stimuli and record the corresponding startle responses. Each test session began with a 5 min acclimatization period in the presence of white noise (70 dB), which continued throughout the session. Six successive pulse-only trials (120 dB for 40 ms) were then presented to calibrate the apparatus, followed by 12 pulse-only trials (120 dB for 40 ms), 42 null trials (no stimulus), and ten prepulse + pulse trials in a pseudorandom order with an average intertrial interval of 7 s (range, 3–12 s). The prepulse + pulse trials consisted of a 20 ms prepulse at one of three different intensities (73, 76, or 82 dB), followed by a 100 ms interval and then the startle pulse (120 dB for 40 ms). The test session lasted for a total of 15 min. Prepulse inhibition score for each prepulse level (PPI73, PPI76, and PPI82) is expressed as % prepulse inhibition (PPI), defined as (1 − (mean startle amplitude in prepulse + pulse trials/mean startle amplitude in pulse-only trials)) × 100. Mean PPI score (PPIm) is defined as [1 − (mean startle amplitude at 73 dB + mean startle amplitude at 76 dB + mean startle amplitude at 82 dB)/(3 ∗ mean startle amplitude in pulse-only trials)] [11].

### 2.5. Data Analysis

Four PPI score, were calculated for each animal at both PND 35 and PND56: PPI73, PPI76, PPI82, and PPIm. For each group of animals, the mean (mean ± SEM) was made for each PPI score. The size of each group was 15. A two-way analysis of variance (ANOVA) was used to evaluate KA × FENO (fenofibrate) interaction, followed by Student's *t*-tests between the four groups when the two-way ANOVA was significant. Statistical significance was defined as *P* < 0.05. All statistical tests have been performed with XLSTAT2011. 

## 3. Results

### 3.1. PPI Scores at PND35

**Figure 1:**
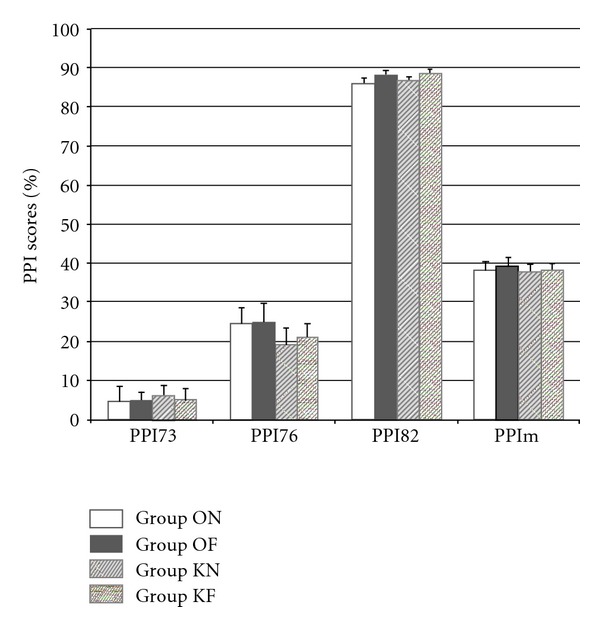
PPI scores (%) at PND35. PPI mean scores at PND35 are comparable between groups. No effect of factors KA or FENO is found with the two-way ANOVA.

The different PPI scores at PND35 are presented in Figure 1.

Means for PPI73 are 6.2% ± 2.5 (KN), 5.1% ± 2.9 (KF), 4.6% ± 3.9 (ON), and 4.8% ± 2.4 (OF). Means for PPI76 are 19.7% ± 3.6 (KN), 21% ± 3.5 (KF), 24.5% ± 4 (ON), and 24.9% ± 4.8 (OF). Means for PPI82 are 85.9% ± 1.3 (ON), 87.9% ± 1.3 (OF), 86.6% ± 0.9 (KN), and 88.7% ± 0.9 (KF). Means for PPIm are 38.3% ± 2.1 (ON), 39.2% ± 2.3 (OF), 37.5 ± 2.1 (KN), and 38.2 ± 1.8 (KF).

Two-way ANOVAs reveal that the effect of factor KA does not explain the variance of PPI scores for PPI73 (*F*1,59 = 0.096, *P* = 0.758), PPI76 (*F*1,59 = 1.167, *P* = 0.285), PPI82 (*F*1,59 = 0.405, *P* = 0.527), and PPIm (*F*1,59 = 0.191, *P* = 0.663). Similarly, the factor FENO does not influence the different PPI scores: PPI73 (*F* = 0.025, *P* = 0.878), PPI76 (*F*1,59 = 0.048, *P* = 0.827), PPI82 (*F*1,59 = 3.2, *P* = 0.8), and PPIm (*F*1,59 = 0.153, *P* = 0.697), and the interaction KA ∗ FENO has no influence either on PPI scores: PPI73 (*F*1,59 = 0.051, *P* = 0.882), PPI76 (*F*1,59 = 0.014, *P* = 0.907), and PPI82 (*F*1,59 < 0.001, *P* = 0.99).

### 3.2. PPI Scores at PND56

**Figure 2:**
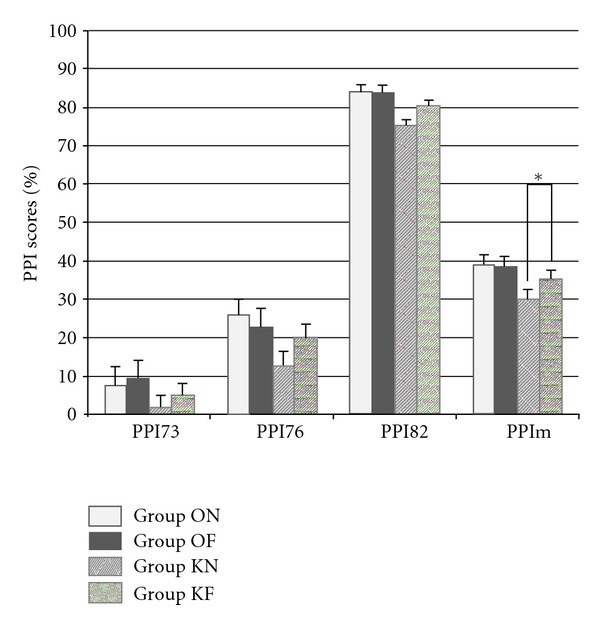
PPI scores (%) at PND56. All PPI mean scores are lower in group KN than in control groups (ON and OF). PPI mean scores in group KF tend to be intermediate between control groups (ON and OF) and group KN. *P < 0.5%.

The different PPI scores at PND56 are presented in Figure 2.

Means for PPI73 are 7.4% ± 2.9 (ON), 9.7% ± 4.4% (OF), 1.77% ± 2.1 (KN), and 4.95% ± 2.7 (KF). Means for PPI76 are 25.9% ± 2.6 (ON) and 22.7% ± 2.4 (OF), 12.6% ± 3.2 (KN) and 20.2% ± 3.2 (KF). Means for PPI82 are 84% ± 1.4 (ON), 83.8% ± 1.3 (OF), 75.4% ± 3.1 (KN), and 80.6% ± 1.8 (KF). Means for PPIm are 39.1% ± 2.7 (ON), 38.8% ± 2.1 (OF), 29.9 ± 1.6 (KN), and 35.3% ± 1.9 (KF). 

A significant effect of factor KA is found in two-way ANOVAs for PPI76 (*F*1,59 = 4.28, *P* < 0.5), PPI82 (*F*1,59 = 8.7,*P* < 0.01), and PPIm (*F*1,59 = 9.6, *P* < 0.01), but not for PPI73 (*F*1,59 = 2.7, *P* = 0.1). No specific effect is found for factor FENO: PPI73 (*F*1,59 = 0.76, *P* = 0.39), PPI76 (*F*1,59 = 0.35, *P* = 0.56), PPI82 (*F*1,59 = 1.5, *P* = 0.22), and PPIm (*F*1,59 = 1.5, *P* = 0.22). The interaction FENO ∗ KA does not reach significance in two-way ANOVAs: PPI73 (*F*1,59 = 0.02, *P* = 0.8), PPI76 (*F*1,59 = 2.1, *P* = 0.16), PPI82 (*F*1,59 = 1.8, *P* = 0.19), and PPIm (*F*1,59 = 1.95, *P* = 0.17).

Student's *t*-tests comparing means between group KN and group KF reveal a significant difference for PPIm scores (*P* < 0.5) but not for PPI82 scores (*P* = 0.154).

## 4. Discussion

First, our study has confirmed in Wistar rat strain what had been previously reported in Long-Evans rats, that is, that KA injection at PND7 could induce PPI disruptions at PND56 that remain undetectable before puberty (PND35) [12]. Furthermore, the effect of KA on PPI seems even more marked in Wistar strain than in Long-Evans, with a clear significant effect in the ANOVAs for both PPI82 (*P* < 0.01) and PPIm (*P* < 0.01). This reinforces the relevance of this method as a neurodevelopmental model relevant with schizophrenia. It should be interesting in forthcoming works to test other rat and mice strains.

Secondly, results at PND56 show a disease-modifying action of fenofibrate on the KA-induced PPI disruption. Results at both PND35 and PND56 show that fenofibrate by itself has no direct action on PPI. On the contrary, fenofibrate appears to correct PPI scores in KA-injected rats, which is in favor of a disease-modifying effect.

The difference between group KN and group KF is visible for all PPI scores, especially for PPI82 and PPIm (see Figure 2). While these differences are not significant in post hoc ANOVA testing, *t*-test reaches significance for PPIm (see Figure 2).

In our study, fenofibrate shows a disease modifying effect on KA-induced PPI disruptions. KA triggers oxidative stress via glutamate KA receptors in the rat brain [21]. Glutamate-related oxidative stress is a form of chemical insult that results in apoptotic processes in neurons [22]. Around PND7 in rat, such lesions are responsible for the occurrence, only at adult age, of disruptions in the mesolimbic dopamine pathway [9]. This results in behavioral anomalies including hyperactivity and reduction in PPI [23].

With a fenofibrate treatment, PPI anomalies are reduced at adult age. Two hypotheses can be made about this disease-modifying effect. The first one is to consider that fenofibrate exerts a neuroprotective action by downregulating the oxidative injuries induced by KA. In so doing, fenofibrate allows to preserve the normal development of PPI-related neurocircuitry. This would suppose, however, that KA triggers oxidative stress chain reactions that persist across the weeks following the initial lesion. While long-term oxidative stress mechanisms have been hypothesized in schizophrenia [24], it has never been proven in neurodevelopmental models of schizophrenia, and notably in this specific model.

Another possible mechanism that could explain the disease-modifying effects of fenofibrate involves direct regulation in cerebral receptors. Indeed, it has been recently proven that the activation of PPAR*α* receptors in the brain could reduce the activity of dopamine neurons [25]. Dopamine activity at adult age is increased by the KA lesion, and PPI disruptions are related to enhanced dopamine transmission [23]. The disease-modifying action of fenofibrate that is reported here could then fit with a dopamine-correction mechanism. In our study, fenofibrate treatment has been continuous from weaning. Its effect on PPI scores could then have been different if the treatment had been stopped somewhile before tests.

Forthcoming research will have to be precise for this model, notably by the mean of histological works, whether the cerebral lesion induced by KA at PND7 consists in a focal oxidative lesion that does not last but diverts the normal course of subsequent neural development, or on the contrary, whether KA triggers chain reactions that keep lesions going long after the initial lesion. Histological samplings could also be precise whether fenofibrate acts by exerting neuroprotective action on oxidative stress processes. If no oxidative stress evidence is observed, then it would be worthy of consideration to look after modifications in the activity of dopamine neurons.

## 5. Conclusion

Postweaning fenofibrate treatment partially reverses postpubertal alterations triggered by KA-induced oxidative lesion at PND7. This could usher new therapeutic perspectives in both prodromal and early phases of schizophrenia. Fenofibrate, which is for being quite a safe treatment, could be tested in young patients, with the perspective of improving the pejorative outcome observed during the first years of schizophrenia. But before that, it remains to be clarified by which mean fenofibrate reduces KA-induced PPI disruptions. Histological samplings could bring more serious argument for hypothesizing that fenofibrate acts by neuroprotection.
